# 

**Published:** 1830-01-01

**Authors:** 


### MEDICO-CHIRURGICAL

### REVIEW,

AND

### JOURNAL

OF

### PRACTICAL MEDICINE.

*(NEW SERIES.)*

VOLUME TWELVE,

[1st of OCTOBER, to 31st of MARCH,]

1830.

VOL. XVI. of ANALYTICAL SERIES.

EDITED BY JAMES JOHNSON, M.D.

*&c. &c. &c.*

Surgeon General's Office LIBRARY 57451 Washington

LONDON:

PUBLISHED BY S. HIGHLEY, 174, FLEET STREET,

AND WEBB STREET, ST. THOMAS'S HOSPITAL,

And all other Booksellers.

WI
ME75T

PRINTED BY G. HAYDEN,

Little College Street, Westminster.

### BIBLIOGRAPHICAL RECORD;

OR,

*Works received for Review from the 25th September to the 25th December*, 1829.

1. Elements of General Anatomy, containing an Outline of the Organization of the Human body. By R. D. Grainger, Lecturer on Anatomy and Physiology. Octavo, pp. 526. Highley, London, 1829.

2. A Manual of General Anatomy; or a concise Description of the Primitive Tissues and Systems which compose the Organs in Man. By A. L. J. Bayle, D. M.P. &c., and H. Holland, D.M.P. &c. Translated from the French by Henry Storer. 18mo. pp. 318. Wilson, London, 1829.

☞ *We have looked over this little Manual and think it extremely well calculated for the student, to whom we can strongly recommend it. It it an excellent addition to the common Dissecting Manual.*

3. A Treatise on Neuralgic Diseases, dependent on Irritation of the Spinal Marrow and Ganglia of the Sympathetic Nerve. By Thos. Pridgin Teale, Member of the Royal College of Surgeons, London; senior Surgeon to the Leeds Public Dispensary, &c. Octavo, pp. 120, Highley, London, 1829.

4. Lectures on Anatomy, interspersed with Practical Remarks; vol. I. By B. B. Cooper, F.R.S. Surgeon of Guy's Hospital, Lecturer on Anatomy, &c. Octavo, pp. 310. Longman, London, 1829. With Plates, 15s.

5. An Account of the Mode of performing the Lateral Operation of Lithotomy; with Illustrations. By Edward Stanley, Assistant Surgeon, and Lecturer on Anatomy and Physiology at Saint Bartholomew's Hospital. Quarto, bds. pp. 23, 7 Plates. Longman, London, 1829.

6. A new Method of treating Burns and Scalds and certain Cutaneous Eruptions. By Mich. C. Ward, M.D. &c. Duodecimo, stitched, pp. 83. Manchester, 1829.

7. A Letter to Lord Robert Seymour, with a Report of the Number of Lunatics and Idiots in England and Wales. By Sir Andrew Halliday, R.H. and M.D. &c. 8vo, stitched, pp. 88. London, 1829.

8. Anatomy of the Human Bones, Joints, and Ligaments; arranged in eight Tables, for the Use of Students. Royal 8vo, stitched. Burgess and Hill, London, 1829, price 1s 6d.

9. A Practical Treatise on Diseases of the Genitals of the Male, with a preliminary Essay on the History, Nature, and general Treatment of Lues Venerea. By By John Maddox Titley, M.D. Octavo, pp. 403. London, 1829.

10. Notions of the Nature of Fever and of Nervous Action. By William Forrester Bow, M.D. &c. Octavo, pp. 100. London, Longman, 1829.

11. The Morbid Anatomy of the Gullet, Stomach, and Intestines. By Alex. Monro, M.D. F.R.S. E. Professor of Anatomy and Surgery in the University of Edinburgh, &c. Second Edition, 8vo, pp. 524, six Plates. Edinburgh, 1829, price 20s.

☞ *A much improved edition of an excellent work.*

---

12. An Introduction to Medical Botany, illustrated with coloured Figures. By THOMAS CASTLE, F.L.S. Member of the College of Surgeons, &c. 18mo. pp. 172, 3 Plates. London, 1829, 5s.

☞ *An indispensable work for Students.*

---

13. Letters to the Royal College of Physicians on their Constitution and Charter; with prefatory Observations to His Grace the Duke of Wellington. By Sir ARTHUR BROOKE FAULKNER, Fellow of the Royal College of Physicians. Octavo, stitched, pp. 48. London, 1829.

---

14. Hospital Facts and Observations illustrative of the Efficacy of the New Remedies, Strychnia, Brucia, Morphia, Veratria, &c. in several Morbid Conditions of the System, &c. &c. By JAMES LOMAX BARDSLEY, M.D. Physician to the Manchester Infirmary, Dispensary, Fever Wards, Lunatic Hospital, and Asylum, &c. Octavo, pp. 223. Burgess and Hill. London, 1830.

---

15. Popular Illustrations of Medicine. By SHIRLEY PALMER, M. D. 8vo, pp, 396. London, 1829.

---

16. Elements of Practical Chemistry, comprising a Series of Experiments in every Department of Chemistry, with Directions for performing them, and for the Preparation and Application of the most important Tests and Re-agents. By DAVID BOSWELL REID, Experimental Assistant to Dr. Hope, &c. 8vo, pp. 511. Maclachlan and Stewart, Edinb. 1829.

---

17. Elements of Physics, or Natural Philosophy, General and Medical, explained independently of Technical Mathematics. In two volumes, Vol. II. Part I. Comprehending the Subjects of Heat and Light. By NEIL ARNOTT, M.D. of the Royal College of Physicians. 8vo, pp. 320. London, Longman, 1829.

---

18. On the Adaptation of Various Parts of the Town of Hastings, as Places of Residence for Invalids in different States of Disease. By WILLIAM HARWOOD, M.D. 12mo, stitched, pp. 40. London, 1829.

☞ *A very useful little pamphlet for those invalids who resort to Hastings. We recommend it to their perusal.*

---

19. An Essay on the Connexion between the Action of the Heart and Arteries and the Functions of the Nervous System, and particularly its Influence in exciting the involuntary Act of Respiration. By JOSEPH SWAN. Octavo, pp. 162, London, 1829.

---

20. On a Morbid Affection of Infancy, arising from Circumstances of Exhaustion, but resembling Hydrencephalus. By MARSHALL HALL, M.D. F.R.S. E. &c. Octavo, stitched, pp. 40. London, 1829.

---

21. A Manual for Students who are preparing for Examination at Apothecaries' Hall. By JOHN STEGGALL, M. D. M.R.C.S. Fourth Edition, with considerable Additions, 8vo, pp. 333. Highley, 1830.

☞ *A very greatly improved edition.*

---

22. A Review of the Doctrine of a Vital Principle, as maintained by some Writers on Physiology. With Observations on the Causes of Physical and Animal Life. By J. C. PRICHARD, M. D. F.R.S. &c. London, 1829, pp. 236.

☞ *We shall make some extracts from this acute and ingenious dissertation in the next number of the Journal.*

---

23. The Muscles of the Human Body, describing their Origin, Insertion, and Use. Arranged in Four Tables, for the Use of Students. London, Burgess and Hill, 1830. Price One Shilling.

☞ *Students will, we think, find these tables very convenient for reference to assist their memory before examinations, or supply the place of more circumstantial details when engaged in dissection.*

---

24. Phrenology Article of the Foreign Quarterly Review. By RICHARD CHENEVIX, Esq. with Notes by G. SPURZHEIM, M.D. Octavo, pp. 70. 1830.

☞ *The Notes are very valuable, and acutely written.*

---

25. A Table of the improved Nomenclature for the Sutures of the Cranium. By H. W. DEWHURST, Surgeon, &c.

☞ *The Nomenclature is decidedly improved in this Table.*

---

26. A Practical Treatise on the Anti-Asthmatic Properties of the Lobelia Inflata, with Directions for its Exhibition, &c. To which is added, an Account of the CHERAYITA HERB, &c. By RICHARD REECE, M.D. Octavo, pp. 40, 1830.

